# Short-term clinical effect of conformal radiotherapy combined with tegafur gimeracil oteracil potassium in treating recurrent esophagus cancer

**DOI:** 10.12669/pjms.325.10632

**Published:** 2016

**Authors:** Yuyan Jiao, Yuzhen Shen, Hua Yan, Yan Liu, Haihua Tan, Jianzhe Li

**Affiliations:** 1 Yuyan Jiao, Oncology Dept.(II), Taian City Central Hospital, Shandong, 271000, China; 2 Yuzhen Shen, Oncology Dept.(II), Taian City Central Hospital, Shandong, 271000, China; 3 Hua Yan, Pediatric Surgery Dept., Taian City Central Hospital, Shandong, 271000, China; 4 Yan Liu, Dermatology Dept., Taian Maternal and Child Health Care Hospital, Shandong, 271000, China; 5 Haihua Tian, Oncology Dept.(II), Taian City Central Hospital, Shandong, 271000, China; 6 Jianzhe Li, Oncology Dept.(II), Taian City Central Hospital, Shandong, 271000, China

**Keywords:** Esophagus cancer, Radiotherapy, Tegafur Gimeracil Oteracil Potassium

## Abstract

**Objective:**

To observe clinical effects of three-dimensional conformal radiotherapy combined with Tegafur Gimeracil Oteracil Potassium chemotherapy in the treatment of patients with recurrent esophagus cancer.

**Methods:**

One hundred and twelve senile patients who suffered from esophagus cancer were selected and randomly divided into two groups, namely, observation group (56 cases) and control group (56 cases). The observation group adopted three-dimensional conformal radiotherapy combined with Tegafur Gimeracil Oteracil Potassium chemotherapy and the control group adopted three-dimensional conformal radiotherapy only.

**Results:**

All patients completed the treatment, with good compliance. Effective rate of the observation group was 82.1%, which was significantly higher than the control group (67.9%), and the difference was statistically significant (P<0.05). Main toxic and side effects of patients of two groups were radiation esophagitis, gastrointestinal reaction, hematologic toxicities and radiative skin reaction. Differences of incidence rates of all types of toxic and side effects were not statistically significant (P>0.05). The one-year and two-year survival rates of patients of the observation group were 80.4% and 53.6%, respectively, while the control group was 55.4% and 30.4%; differences between two groups were statistically significant (P<0.05).

**Conclusion:**

Three-dimensional conformal radiotherapy combined with Tegafur Gimeracil Oteracil Potassium chemotherapy has definite curative effect in treating patients with recurrent esophagus cancer and can improve survival rate of patients, without increasing adverse reaction.

## INTRODUCTION

Esophagus cancer is a common gastrointestinal tumor. It is estimated that mortality of esophagus cancer around world are 300 thousand annually, and the morbidity and case fatality rate are obviously regionally different.^1^,^2^ Morbidity of esophagus cancer of males is higher than that of females, and the age of onset is mostly over 50 years old.^3^ China is a country with high morbidity of esophagus cancer, where about 150 thousand people die of it annually, and with aged tendency of population, constituent ratio of morbidity of senile patients showed an increasing tendency.^4^,^5^

Currently, surgical operation remains to be a major treatment for esophagus cancer, but patients usually die of relapse and metastasis after operation.^6^ Radiotherapy is a main treatment for postoperative relapse and local metastasis of esophagus cancer has. With the update of therapeutic equipment and methods of radiotherapy, radiotherapeutic effects of esophagus cancer increased, but prognosis of it is unsatisfactory.^7^ Therefore, applying concurrent radiochemotherapy scheme in the treatment of patients with recurrent esophagus cancer has become a research hotspot. It has been reported that, oral adminstration of Tegafur Gimeracil Oteracil Potassium combined with radiotherapy could increase curatives effects of esophagus cancer; however, concurrent radiochemotherapy may increase radiotherapy-related adverse reaction of patients.^8^,^9^

The study selected one hundred and thirteen senile patients with postoperative recurrence of esophagus cancer, randomly divided them into three-dimensional conformal radiotherapy group (the control group) and the concurrent radiochemotherapy group (three-dimensional conformal radiotherapy + Tegafur Gimeracil Oteracil Potassium) (the observation group), and analyzed the clinical effects of two groups.

## METHODS

One hundred and twelve senile patients with recurrent esophagus cancer were included and treated by department of radiotherapy of the Taian City Central Hospital from October 2012 to October 2014. All of the patients signed informed consent Forms. It included 76 males and 36 females, aged from 65 to 71 years (average (68±3) years). Karnofsky performance score (KPS) was greater than 70 points. All the patients were confirmed as squamous carcinoma through gastroscope. They were treated with cervical esophagogastrostomy 6 to 36 months earlier. As to postoperative staging, 53 cases were at stage II, and 59 cases were at stage III.

All the patients received postoperative adjuvant chemotherapy for 2 to 4 cycles. Sites of recurrence of 22 cases were at anastomotic stomas, 46 cases were at mediastinal lymph nodes, and 44 cases were at necks and clavicles. All recurrent foci underwent gastroscope and puncture and were confirmed by pathological biopsy. All the patients were with measurable foci and their expected lifetime was equal or greater than three months. Conventional electrocardiogram, cardiac color ultrasound, hematology analysis and liver function detection performed after admission suggested the heart, liver, liver and hepatic functions and blood had no contraindications to radiotherapy and chemotherapy.

The patients were randomly and evenly divided into two groups according to random number table. Patients in the observation group were treated with three-dimensional conformal radiotherapy in combination with Tegafur Gimeracil Oteracil Potassium, and patients in the control group were treated with three-dimensional conformal radiotherapy). Differences of age, gender, pathological pattern and Kamofsky performance score were not statistically significant (P>0.05); hence, the results were comparable.

### Therapies

The control group adopted three-dimensional conformal radiotherapy, and the exposure dose was 1.8Gy/time, five times a week. Radiotherapy was continually performed for six weeks; the total radiological dose was 60Gy. Besides three-dimensional conformal radiotherapy, patients of the observation group orally took 40 to 60 mg of Tegafur Gimeracil Oteracil Potassium chemotherapy, twice each day, for four weeks. Patients received liver protection treatment, vomit stopping treatment and supportive treatment when they were treated with chemotherapy. Patients with level two or higher diarrhea were provided with symptomatic treatment by using smecta, Imodium and etc. Patients with bone marrow suppression were injected with human granulocyte colony stimulating factor (G-CSF).

### Criteria of curative effects

According to response evaluation criteria in solid tumors (RECIST) 1.1, short-term effects were divided into: complete remission (CR), partial remission (PR), stable disease (SD), and progressive disease (PD). Disappearance of measurable foci lasting for one or more months was determined as CR; 30% or more reduction of measurable foci lasting for one or more months was determined as PR, and 30% less reduction of measurable foci was determined as SD; 20% or higher increase of measurable foci or newly developed foci was determined as PR.10 The calculation formula of total effect rate was: total effective rate = CR+PR.

### Observation index

Short-term effects, one-year and two-year survival rates, and adverse reactions of patients in two groups were compared after treatment.

### Statistical analysis

SPSS 20.0 was used for data analysis. Measurement data were processed by t-test, while the enumeration data were tested by chi-square test. If P<0.05, difference was considered to be statistically significant.

## RESULTS

### Comparison of clinical effects between two groups

All patients completed the treatment, with good compliance. Total effective rate of the observation group and the control group was 82.1% and 67.9% respectively. The difference was statistically significant (P<0.05). Details are shown in Table-I.

**Table-I:** Comparison of clinical effects between two groups of patients [N(%)].

*Group*	*CR*	*PR*	*SD*	*PD*	*Effective rate*
Control group(N=56)	3(5.4)	35(62.5)	12(21.4)	6(10.7)	38(67.9)
Observation group(N=56)	11(19.6)	35(62.5)	9(16.1)	1(1.8)	46(82.1)
X^2^	5.87	0.64	0.89	4.36	4.13
P	< 0.05	>0.05	>0.05	< 0.05	< 0.05

### Comparison of toxic and side effects between two groups

Main toxic and side effects of two groups of patients included hematologic toxicities, gastrointestinal reaction and radiative skin reaction. Except for two cases of level 3 reduction of leukocytes, the other cases were at level 1 or 2; gastrointestinal reactions mainly induced nausea, emesis and radiation esophagitis. All the toxic and side effects were reversible, and could disappear or be alleviated through symptomatic treatment or drug withdrawal. There was no chemotherapy-related death in the study. Details are shown in Table-II.

**Table-II:** Comparison of toxic and side effects between two groups of patients

*Group*	*Reduction of leukocytes*		*Reduction of hemoglobin*	*Nausea, Emesis*	*Diarrhea*	*Radiation esophagitis*

	*Level 1-2*	*Level 3*
Control group (N=56)	41	0	9	35	6	32
Observation group (N=56)	44	2	11	47	9	34
X^2^	1.13	2.34	0.39	0.56	0.12	
P	>0.05	>0.05	>0.05	>0.05	>0.05	

### Comparison of survival rate between two groups of patients

The one-year survival rate of the observation group was 80.4% and the two-year survival rate was 53.6%, which were higher than that of the control group (55.4%, 30.4%), and the differences were statistically significant (P<0.05). Details are shown in Table-III.

**Table-III:** Comparison of one-year and two-year survival rates between two groups of senile esophagus cancer patients [N(%)].

*Group*	*One-year survival rate*	*Two-year survival rate*
Control group(N=56)	45(80.4)	30(53.6)
Observation group (N=56)	31(55.4)	17(30.4)
X^2^	4.89	4.56
P	< 0.05	< 0.05

## DISCUSSION

With the improvement of radiotherapy techniques, precision radiotherapy has been widely used in clinical practice. Also, due to the continuous improvement of tolerance to radiotherapy, concurrent radiochemotherapy scheme can be applied to senile patients, and it is gradually becoming the treatment of choice for locally advanced and postoperatively relapsed esophagus cancer.^11^,^12^ Hirose T et al. found that concurrent radiochemotherapy could not be completed by 31% to 34% of esophagus cancer patients due to toxic and side effects.^13^ For those patients who cannot tolerate concurrent radiochemotherapy, they can only adopt radiotherapy alone. However, clinical cure rate of simple and conventional radiotherapy for esophagus cancer with postoperative relapse or regional lymph nodes metastasis is only 60%, and rate of complete is only 5% to 8%.^14^ Besides, control time of radiotherapy alone is shorter, and local failure rate in one year is as high as 48%. In recent years, Tegafur Gimeracil Oteracil Potassium chemotherapy combined with conformal radiotherapy has been found to be effective in enhancing long-term efficacy and increasing three-year survival rate of patients.^15^ Nevertheless, there are few Chinese studies on the application of Tegafur Gimeracil Oteracil Potassium chemotherapy combined with conformal radiotherapy in the treatment of senile esophagus cancer.

Tegafur Gimeracil Oteracil Potassium is a new fluorouracil derivative, containing 3 ingredients, i.e., Tegafur (FT), gimeracil (CDHP) and oteracil (Oxo).^16^ FT is the prodrug of 5- Fluorouracile (5-Fu), which has good oral bioavailability, and can be converted into 5-Fu in living body. CDHP is able to inhibit catabolism of 5-Fu, and thereby significantly improving blood concentration of 5-Fu in tumor tissue and blood.^17^,^18^ Oxo can block phosphorylation of 5-Fu, and it will lead to high distribution concentration in gastrointestinal tissue after drug taking, therefore, the distribution of 5-Fu in gastrointestinal tract will be influenced and then adverse effects of 5-Fu can be reduced.^19^ Compared with 5-Fu, Tegafur Gimeracil Oteracil Potassium can lower influences of drugs on marrow hematopoietic system, especially on neutrophil and thrombocyte, and meanwhile reduce incidence of some symptom of digestive tract such as nausea and diarrhea. Tegafur Gimeracil Oteracil Potassium has the following advantages compared to 5-Fu. First, it is capable of maintaining relatively high blood drug concentration and enhance antitumor activity. Besides, it can obviously reduce toxicity of drugs; In addition, the medication of Tegafur Gimeracil Oteracil Potassium is easy. Li JB et al. found, treating locally advanced esophageal cancer in middle and upper segment of chest with intensity modulated radiation therapy using Tegafur Gimeracil Oteracil Potassium and cis-platinum can result in a high clinical effective rate and mild adverse reaction.^20^ Zhang YX et al. thought that, adopting Tegafur Gimeracil Oteracil Potassium radiotherapy could achieve better curative effect and longer survival time in the treatment of patients with esophagus cancer.^21^

Results of this study showed that, foci of 83.3% of cases in the observation group were stable or reduced, suggesting Tegafur Gimeracil Oteracil Potassium could be a clinically effective and low-toxic drug for patients with recurrent esophagus cancer. In addition, results showed that, the one-year survival rate and the second-year rate of the observation group were 80.4% and 53.6% respectively, which were higher than those of the control group (55.4%, 30.4%), and the differences were statistically significant (P<0.05). As to adverse reactions, although the incidence of gastrointestinal reaction, bone marrow suppression and radiation esophagitis of synchronous chemoradiotherapy group were higher than the radiotherapy group, they could be alleviated by symptomatic treatment such as anti-infection, hormone, granulocyte colony-stimulating factor and nutritional support treatments. All patients successfully completed the treatment plan.

## CONCLUSION

To sum up, compared with radiotherapy alone, three-dimensional conformal radiotherapy in combination Tegafur Gimeracil Oteracil Potassium chemotherapy can significantly improve efficacy and survival rate. Though the therapy increases adverse reactions, they are all tolerable by patients. Therefore, it deserved to be clinically promoted.
